# Bone marrow biopsy can be omitted in the diagnostic workup of CNS lymphoma of DLBCL origin: a population-based retrospective study in the PET-CT era

**DOI:** 10.1007/s00277-023-05282-7

**Published:** 2023-05-29

**Authors:** Jelena Jelicic, Dennis Lund Hansen, Sarah Sand Carlsen, Michael Thorsgaard, Ditte Stampe Hersby, Karina Kannik, Amalie Sofie Eilsø Munksgaard, Thomas Stauffer Larsen, Karen Juul-Jensen

**Affiliations:** 1 Department of Hematology, Odense University Hospital, Odense, Denmark; 2 Department of Hematology Vejle Hospital, Vejle, Denmark; 3 Department of Clinical Research, University of Southern Denmark, Odense, Denmark; 4 Department of Hematology, Zeeland University Hospital, Roskilde, Denmark; 5 Department of Hematology, Aarhus University Hospital, Aarhus, Denmark; 6 Department of Hematology, Copenhagen University Hospital, Rigshospitalet, Denmark; 7 Department of Hematology, Aalborg University Hospital, Aalborg, Denmark

**Keywords:** Diffuse large B cell lymphoma, Central nervous system lymphoma, Bone marrow biopsy, PET-CT

## Abstract

Currently, bone marrow (BM) biopsy (BMB) is recommended in the initial staging of patients with the presumed primary central nervous system (CNS) lymphoma (PCNSL). However, the added value of BMB in the era of positron emission tomography *(*PET-CT) has been challenged in other lymphoma subtypes. We analyzed BM findings in patients with biopsy-proven CNS lymphoma and a negative PET-CT scan for disease outside CNS. A comprehensive Danish population-based registry search was performed to identify all patients with CNS lymphoma of diffuse large B cell lymphoma (DLBCL) histology with available BMB results and staging PET-CT without systemic lymphoma. A total of 300 patients fulfilled the inclusion criteria. Of them, 16% had a previous history of lymphoma, while 84% were diagnosed with PCNSL. None of the patients had DLBCL in the BM. A minority (8.3%) had discordant BMB findings, mainly low-grade histologies that did not influence treatment choice in any case. In conclusion, the risk of overlooking concordant BM infiltration in patients with CNS lymphoma of DLBCL histology and negative PET-CT scan is negligible. As we did not find any patient with DLBCL in the BMB, our results suggest that BMB can be safely omitted in the diagnostic workup in patients with CNS lymphoma and a negative PET-CT.

**Supplementary Information:**

The online version contains supplementary material available at 10.1007/s00277-023-05282-7.

## Introduction

Primary central nervous system (CNS) lymphoma (PCNSL) is a rare and aggressive disease restricted to the brain, spinal cord, cerebrospinal fluid (CSF), and eyes [1]. It accounts for about 7% of all malignant primary brain tumors and 4–6% of extranodal lymphomas [1, 2]. Histopathologically, more than 90% of the PCNSLs are found to be diffuse large B cell lymphomas (DLBCL). In contrast, T cell lymphoma, Burkitt lymphoma, and low-grade lymphomas are rare [1]. The prognosis of patients with PCNSL is poor, and 60% will eventually relapse [3]. However, systemic relapse of CNS lymphoma is rare and occurs in only approximately 2.7% of patients diagnosed with PCNSL [3]. Although long-term survival is possible in some patients with isolated CNS relapse of systemic lymphoma, the outcome is generally poor, with a median survival of 1.6 years [4]. In patients with systemic non-Hodgkin lymphoma (NHL), CNS relapse either confined to the CNS or as part of systemic recurrence often represents an end-stage disease with a very poor outcome and current treatment strategies [4]. Approximately 5% of patients with systemic NHL experience CNS relapse, with only 1% having an isolated CNS relapse with no evidence of lymphoma outside the CNS at the time of relapse [4].

In patients who develop neurologic deficits, diagnostic imaging is needed [1]. Stereotactic biopsy of the brain lesion is the procedure of choice if lymphoma is suspected. Additionally, a vitrectomy should be performed in case of ocular involvement [1]. The International PCNSL Collaborative Group recommends performing baseline staging to determine the extent of disease, including both contrast-enhanced magnetic resonance imaging (MRI) of the brain and the spine in case of spinal symptoms, as well as ophthalmologic and CSF (cerebrospinal fluid) evaluation [5]. To exclude the presence of disease outside of CNS, a fluorodeoxyglucose (FDG) positron emission tomography (PET-CT) scan, and a bone marrow (BM) biopsy (BMB) should be performed [1]. Before the International PCNSL Collaborative Group introduced current consensus recommendations, the importance of a complete baseline staging had widely been discussed in the literature [6, 7]. Current recommendations of baseline evaluation with BMB are primarily based on studies published in the pre-PET-CT era. These studies challenged previous publications suggesting limited workup without imaging and BMB in patients with presumed PCNSL due to the low risk of systemic lymphoma [8]. However, selected studies reported that 4–12% had a change in treatment strategy due to comprehensive workup [2, 6, 9]. One of the most significant studies performed in the pre-PET-CT era analyzed 128 patients diagnosed between 1975 and 1994 initially thought to have PCNSL and found systemic lymphoma in 3.9% of cases, with only one patient with BM involvement [7]. Compared to PET scans, a computed tomography (CT) scan is known to underestimate the clinical stage of the disease in patients with aggressive lymphoma and entails an inherent risk of missing a diagnosis of systemic lymphoma with secondary CNS involvement [10]. Recently, several studies questioned the necessity of BMB in patients with presumed PCNSL due to the low risk of systemic lymphoma involvement in these patients [2, 11]. However, routine BMB is still a part of the staging setting and may hypothetically be omitted, provided FDG-PET CT demonstrates no suspicion of systemic disease.

This study investigated the added value of BMB in patients with biopsy-proven CNS lymphoma of DLBCL histology and whether BMB can be omitted in the diagnostic workup in patients with a negative PET-CT.

## Methods

We performed a retrospective multicenter study on all CNS lymphoma-treating medical centers in Denmark. Patients diagnosed from January 2002 to January 2020 were eligible for inclusion in the current study.

### Search criteria

Potential candidates for the study were identified from the Danish Pathology Register (DPR) by 1) diagnosis (DLBCL) as well as observational diagnosis and 2) site of biopsy (central nervous system, meninges, or eyes). When potential candidates were identified in the DPR, electronic medical records of each patient were reviewed by each center where the patient was diagnosed in order to confirm the diagnosis. The DPR is a comprehensive national database of all pathology reports archived in a nationwide registry linked to the patient’s unique personal identification number (CPR number) [12]. The Danish Civil Registration System ensures the unique identification of all inhabitants in Denmark. The coverage of the DPR is almost 100% because the registration is performed electronically as part of the electronic sign-out of a pathology report [12].

When potential candidates were identified, electronic medical records of each patient were reviewed by an expert hematologist or hematologist in training at each of the participating centers responsible for diagnostic workup and treatment of individual patients. Relevant data from eligible patients were collected and managed anonymized using the electronic data capture system RedCap, Vanderbilt University.

### Inclusion/exclusion criteria

Patients were included in the final study cohort if they fulfilled all the following inclusion criteria: a) biopsy-proven lymphoma of DLBCL type restricted to CNS compartments (parenchyma, meninges, corpus vitreum, retina, and/or cerebrospinal fluid and b) available reports on both staging PET-CT scan and BMB. Patients with a history of prior systemic lymphoma but recurrence restricted to CNS were included.

Patients were excluded if they a) were diagnosed with CNS lymphoma other than DLBCL, b) were diagnosed with systemic DLBCL with CNS involvement, and c) did not have both a BMB and/or PET-CT as part of diagnostic workup (unavailable results on PET-CT and BMB in the electronic medical record).

Clinical data retrieved from eligible patients included the following: age at diagnosis, gender, involvement of lymphoma in BM (both concordant and discordant), involvement of systemic lymphoma on PET-CT, tumor localization (supratentorial, infratentorial, vitreoretinal, and CSF), and number of lesions (multifocal and isolated).

### PET-CT data

All fluorodeoxyglucose (FDG) PET-CT scans were performed as whole-body scans (vertex or midbrain to upper thigh) after a 4–6-hour fast. Emission data were acquired for 2 to 5 minutes per bed position starting about 60 min after intravenous injection of the radiotracer (activity of 3.5–4.5 MBq/Kg) with glucose level lower than 150 mg/dL.

PET-CT studies were obtained on the following scanner types: Philips Gemini TF, GE Discovery (LS, VCT, STE, RX, and MI5) (GE Healthcare, Milwaukee, WI), and Siemens Biograph (TruePoint 16, TruePoint 40, TruePoint 64, Vision 600, Quadra) (Siemens Healthcare, Erlangen, Germany). Quality control procedures were performed at regular intervals for all scanner types, with strict adherence to local protocols and international accreditation criteria.

In general, the CT components of the PET-CTs were all contrast-enhanced. PET-CT studies were assessed visually using Deauville criteria in case that was relevant according to clinical guidelines [13]. The PET images were analyzed by nuclear medicine physicians with experience in PET-CT and lymphoma. All written PET-CT reports were retrieved and carefully reviewed by data collectors in order to evaluate peripheral involvement according to clinical practice.

All procedures followed were performed in accordance with the ethical standards of the responsible committee on human experimentation (institutional and national) and with the Helsinki Declaration of 1975, as revised in 2008.

### Statistical analysis

The analyzed population was described using basic summary statistics. Percentages and frequencies were used for categorical measures. Medians, interquartile ranges, and ranges were used for continuous variables and compared with the Mann–Whitney test [14]. The probability of the highest possible risk of BM involvement was investigated with univariate Bayesian regression, using non-informative and the results from Margold et al. as informative priors [2].

## Results

**Fig. 1:**
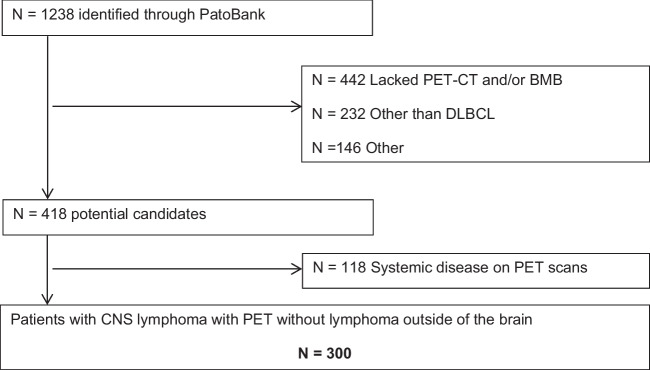
Flowchart of the selection process of patients with diffuse large B cell lymphoma (DLBCL) in the central nervous system (CNS) with available bone marrow biopsy (BMB) in PET-CT (positron emission tomography/computed tomography) era

A total of 1238 patients were identified through the initial search in the Pathology Registry (Fig. 1). The main reasons for exclusion were the following: a) not performed PET-CT scanning, BMB, or both as part of diagnostic workup, b) histological diagnosis other than DLBCL, and c) unavailability of medical records for patients diagnosed in the early 2000s. Of 418 potential candidates, additional 118 patients were excluded because of lymphoma outside of CNS on PET-CT scans. Finally, a total of 300 patients with DLBCL histology in the CNS and no systemic lymphoma on PET-CT were identified.

Of 300 patients, 252 (84%) were diagnosed with PCNSL, and 48 (16%) had a history of prior lymphoma (referred to in the text as isolated CNS relapse of systemic lymphoma). The median age was 68 years (IQR, 6.5–71.0) (Table 1). There was no difference in age between the patients with PCNSL and those with the isolated CNS relapse of systemic lymphoma (median age 68 years vs. 65.5 years, *p* = 0.12). There was a male predominance (180 patients, 60%). Most patients (248 patients, 82.7%) had supratentorial brain involvement, while 13.7% had infratentorial involvement. Vitreoretinal involvement was detected in 12 patients, of whom nine were diagnosed with vitreoretinal lymphoma alone. DLBCL cells in CSF were detected in 33 patients (11%). In 10 patients (3.3%), MRI scans detected leptomeningeal involvement. (Table 1). Although we did not register the duration and dose of corticosteroid administration, we noted the number of patients who received corticosteroids at some point during the diagnostic period. Corticosteroids were administered to 254 of 289 patients (87.9%), excluding 11 patients whose information on steroid treatment during the diagnostic period was unavailable. Regarding steroid administration, there was no difference between PCNSL patients (217 of 243 patients, 89.3% (95% CI, 84.7–92.9%)) and patients with isolated relapse in CNS (37 of 46 patients, 80.4% (95% CI, 66.1–90.6%), *p* = 0.091). Only 1 of 8 patients with primary vitreoretinal DLBCL received corticosteroids during the diagnostic period.

**Table 1:** Clinical characteristics of patients and localization of diffuse large B cell lymphoma (DLBCL) in the central nervous system (CNS)

	All patients (*n* = 300)	PCNSL (*n* = 252)	Isolated CNS relapse of systemic lymphoma (*n* = 48)
Age median, years (IQR)	68.0 (60.5, 74.0)	68.0 (61.0, 74.0)	65.5 (58.0, 71.5)
Male/female, *n* (%)	180 (60.0)/120 (40.0)	148 (58.7)/104 (41.3)	32 (66.7)/16 (33.3)
Localization, *n* (%)			
Supratentorial	248 (82.7)	211 (83.7)	37 (77.1)
Infratentorial	41 (13.7)	36 (14.3)	5 (10.4)
Vitreoretinal*	12 (4.0)	11 (4.4)	1 (2.1)
CSF**	33 (11.0)	21 (8.3)	12 (25)
Leptomeningeal involvement on MRI	10 (3.3)	6 (2.4)	4 (8.3)

*CI* confidence intervals, *CNS* central nervous system, *CSF* cerebrospinal fluid, *IQR* interquartile range, *MRI* magnetic resonance imaging, *PCNSL* primary central nervous system lymphoma

*Both primary vitreoretinal lymphoma and in combination with other CNS sites

**Both isolated CSF involvement and in combination with other CNS sites

### Bone marrow biopsy results

#### Concordant findings

No patients with negative PET-CT scans were found to have concordant DLBCL in BMB. In order to assess the potential risk of an event that did not occur in the current study population, we performed a Bayesian regression using non-informative and the results from Margold et al. as informative priors [2]. With this information alone in our cohort of 300 patients, the risk of BM involvement is 0.98% or less, with a 95% probability. Using the informative prior, the risk is 0.77% or less with a 95% probability. Only looking at the cohort of PCNSL (excluding patients with a history of prior lymphoma), the risk of BM involvement is with 95% probability less than 1.2% with uninformative prior and less than 0.85% with informative priors. No appropriate study for this analysis could be found as a referent for the cohort of patients with the isolated CNS relapse of systemic lymphoma, possibly due to the rarity of this entity. Therefore, only the uninformative prior was applied, giving a risk of less than 5.85% with a 95% probability. Kernel density plots of the probability are depicted in the online supplementary Figure 1.

#### Discordant findings

The vast majority had normal BMB (275, 91.7%). Various discordant pathological findings in BM were registered among 25 patients (8.3%), including low-grade lymphoma not otherwise specified in 5 patients, chronic lymphocytic leukemia in 3 patients, lymphoplasmacytic lymphoma in 3 patients, and marginal zone lymphoma in 2 patients. One patient was diagnosed with monoclonal gammopathy of undetermined significance (MGUS). Another with a medical history of smoldering myeloma had this diagnosis confirmed in the BMB. Additionally, nine patients had monoclonal B cells in the BMB, while one was diagnosed with T-large granular lymphocytic leukemia. More detailed information on discordant findings in the BMB is presented in Table 2.

**Table 2.** Clinical and histopathological findings of patients with diffuse large B cell lymphoma (DLBCL) in the central nervous system (CNS) and discordant findings in the bone marrow

No.	Gender	Type of CNS lymphoma	BM findings	Flow Cytometry	Infiltration in % on flow cytometry/(ICH*)	Final diagnosis
01	F	PCNSL	MBL clone with CLL profile	Clonal B-lymphocytes	0.8	MBL
02	M	PCNSL	CLL	CLL	40.0	CLL
03	F	PCNSL	CLL-like clone with an increased number of prolymphocytes	Clonal B-lymphocytes with CLL profile	23.0	CLL
04	F	PCNSL	MBL clone with non-CLL profile	Clonal B-lymphocytes with FL profile	0.3	MBL
05	M	rCNSL	LPL	Clonal B-lymphocytes with LPL profile	NA/(2.0–3.0)	MBL
06	F	PCNSL	T-LGL	Polyclonal B-lymphocytes/T-lymphocytes positive for CD2, CD3, CD5, CD8 and CD28	0.6%/(NA) 67.0%/(NA)	T-LGL
07	M	PCNSL	MBL clone with non-CLL profile	Clonal B-lymphocytes	1.0	MBL
08	M	PCNSL	MBL clone with CLL profile	Clonal B-lymphocytes	0.4	MBL
09	M	PCNSL	LPL	Not performed	NA/(50.0)	LPL
10	M	PCNSL	Not MCL, DLBCL, or CLL, very low Ki-67	Not performed	NA	Low-grade B cell lymphoma
11	M	PCNSL	Hypercellular BM, no lymphoma cells,	Clonal B lymphocytes with CD19+/CD20+/CD5-/kappa+ phenotype	6.0	MBL
12	M	rCNSL	LPL	Not performed	NA	LPL
13	F	PCNSL	MM	Clonal plasma cells	24.0	SMM
14	F	rCNSL	Low malignant B cell lymphoma	Not performed	NA	Low-grade B cell lymphoma
15	F	PCNSL	MBL with CLL profile	Clonal B-lymphocytes	0.1	MBL
16	F	PCNSL	MBL with CLL profile	Clonal B-lymphocytes	0.2	MBL
17	F	PCNSL	MZL	Not performed	NA/(30.0)	MZL
18	M	rCNSL	IgM MGUS	No LPL in BM	NA	MGUS
19	F	PCNSL	Suspected low malignant B cell lymphoma	No diagnostic criteria fulfilled	NA	Low-grade B cell lymphoma
20	M	PCNSL	Suspected low malignant B cell lymphoma	No diagnostic criteria fulfilled	NA	Low-grade B cell lymphoma
21	F	PCNSL	LPL/Waldenström	Clonal B lymphocytosis	11.0	MBL
22	F	PCNSL	MBL with CLL-like profile and low malignant B cell lymphoma	Kappa B cells without characteristic phenotype/kappa B cells with CLL-like profile.	14.0/2.0	Low-grade B cell lymphoma/MBL
23	M	rCNSL	CLL	CLL/0.8% kappa clonal B cells	20.0	CLL
24	F	PCNSL	Low malignant B cell lymphoma, likely MZL	Not performed	NA/(10.0)	MZL
25	M	PCNSL	LPL	Not performed	NA	LPL

*BM* bone marrow, *CLL* chronic lymphocytic leukemia, *CNSL* central nervous system lymphoma, *DLBCL* diffuse large B cell lymphoma, *F* females, *FL* follicular lymphoma, *I* immunohistochemistry, *LPL* lymphoplasmacytic lymphoma, *M* male, *MBL* monoclonal B-lymphocytosis, *MGUS* monoclonal gammopathy of undetermined significance, *NA* not applicable, *MZL* marginal zone lymphoma, *rCNSL* isolated relapse of lymphoma in CNS, *SMM* smoldering multiple myeloma, *T-LGL* T-large granular lymphocytic leukemia

*Bone marrow infiltration assessed by immunohistochemistry in selected cases

Patients with discordant BMB findings (*n* = 25) were older compared to patients with isolated CNS lymphoma (*n* = 275), being either primary (*n* = 20) or secondary (n = 5) (74.0 years (IQR: 68.0–78.0) vs. 67.0 years (IQR: 60.0–74.0), *p* = 0.003). No difference in gender distribution (male/female ratio; 167/108 vs. 13/12, *p* = 0.40) was observed between these groups. Similar results were observed when PCNSL (*n* = 252) patients were analyzed alone. Patients without discordant findings in the BMB and no history of prior lymphoma were younger (*n* = 232, 67.5 years (IQR: 61.0–74.0)) than the patients with discordant findings (*n* = 20, 73.0 years (IQR: 68.0–78.0), *p* = 0.01). There was no difference between these two populations regarding gender (*p* = 0.24).

Patients with isolated CNS relapse of systemic lymphoma had a higher prevalence of discordant findings in the BMB (5/48, 10.4%) than those with PCNSL (20/252, 7.9%). However, the difference was not statistically significant. Among these 48 patients with isolated DLBCL relapse in the CNS, three patients had low-grade lymphoma, and two were diagnosed with MGUS and MBL in the BMB, respectively.

## Discussion

This study aimed to investigate the diagnostic value of BMB as part of routine staging in patients with CNS lymphoma of DLBCL type with no signs of systemic lymphoma outside of CNS on PET-CT scans. Currently, BMB is recommended as a part of initial staging in patients with presumed PCNSL. We analyzed BMB findings in the absence of systemic lymphoma on FDG-PET scans to assess whether patients with presumed CNS lymphoma and no perceived systemic lymphoma on FDG-PET scans could be spared unnecessary invasive BMB. A comprehensive search was performed to identify all patients with CNS lymphoma. We included 300 patients with CNS lymphoma of DLBCL histology and no signs of lymphoma outside of the CNS. Of them, 252 patients were diagnosed with PCNSL, while 48 had isolated CNS relapse of systemic lymphoma. We did not find any patient with concordant lymphoma in the BM. This supports our hypothesis that BMB can be safely omitted in the diagnostic workup of PCNSL of DLBCL histology. However, we identified 25 patients with discordant findings that did not influence the treatment approach [2]. In line with the literature data, these patients were slightly older than those with normal BMB (median age 67 vs. 74 years) [2].

Systemic dissemination of PCNSL has been reported in up to 12% of patients with presumed PCNSL when systematic diagnostic procedures were performed [6, 7]. The most recent study performed on a large cohort of patients with biopsy-proven CNS DLBCL reported an overall detection rate of systemic disease of 2.6% (27/1043) when CT and/or whole-body PET-CT was performed [15]. Treatment adjustments were made in 74% of these patients [15]. Although precise data on BMB were unavailable, PET-CT was performed in 81.9% of patients. Twenty-five of 27 patients with systemic lymphoma underwent whole-body PET-CT, and no one was diagnosed with a disease in the BM [15]. However, few cases with BM involvement as the only systemic manifestation of CNS lymphoma have been reported in the literature [6, 7]. Most studies investigating the extent of systemic dissemination of PCNSL were performed before the routine implementation of PET-CT as part of staging. The importance of performing PET-CT as part of staging procedures to exclude lymphoma outside of CNS and secondary malignancies in PCNSL has been established throughout the years [16–18]. Several recent studies have questioned the added value of BMB at diagnosis in patients with presumed PCNSL, as the introduction of PET-CT scans has already changed the diagnostic approach in some lymphomas [2, 11, 16].

The first studies that evaluated the role of PET-CT in patients with lymphoma concluded that PET-CT is superior to ordinary CT and is equivalent to BMB in detecting BM involvement [10]. The high sensitivity of PET-CT for BM involvement has questioned the continued use of BMB in several other common B cell malignancies leading to the exclusion of BMB as part of staging in Hodgkin lymphoma based on retrospective studies [16]. Regarding systemic DLBCL, several studies reported a high negative predictive value for detecting BM involvement [10, 19, 20]. According to data from 930 patients with aggressive lymphoma from PETAL and OPTIMAL > 60 trials, BM infiltration was found by PET-CT in 20% of patients but in only 9% by BMB [21]. Moreover, patients with a positive BMB had other factors consistent with advanced-stage or poor prognosis, and consequently, BMB findings did not change the treatment strategy [21]. This is in accordance with clinical practice guidelines for aggressive lymphomas recommending that PET-CT may replace BMB in these patients [16]. However, a BMB should be considered to identify involvement by discordant histology if relevant for patient management [16, 22]. Individual studies have discussed the role of BMB in PCNSL patients in the era of PET-CT. Albano et al. analyzed 46 patients with biopsy-proven brain lymphoma and detected extracranial hypermetabolic lesions in BM in one patient who had confirmed BM disease by histopathological analysis [23]. Bertaux et al. found 3 out of 130 PCNSL patients with pathological uptake in BM by PET-CT at initial staging. However, only one had confirmed BM involvement by BMB [24]. In this study, 95% of patients had DLBCL PCNSL, including a single patient with BM involvement [24]. These findings suggest that PET-CT scans can upstage patients and change subsequent clinical management. However, there is a possibility of missing low-volume diffuse involvement of 10 to 20% of the marrow [16]. Approximately 5–10% of patients with systemic DLBCL lymphoma are diagnosed with BM involvement, and 5–12% with discordant findings [2]. Regarding patients with PCNSL, Margold et al. recently published a retrospective analysis of 392 patients with presumed PCNSL, of whom 320 had available BMB results. The authors found 23 pathologic results in the BM, with 22 samples showing discordant BMB findings other than the histology of brain lymphoma. Only one harbored the same lymphoma in the brain and BMB with an early progression and OS of only seven months, supporting that concordant lymphoma in BM is associated with poor prognosis. However, the study did not include imaging data. In line with the literature data, the study concluded that the frequency of concordant CNS and BM lymphoma with no systemic involvement is exceedingly low [2]. Regarding the other 22 discordant findings in BM, most had low-grade lymphoma in the BM (*n* = 12). In contrast, the other showed B cell proliferation but no proof of lymphoma (*n* = 5), monoclonal B cells (*n* = 3), or abnormalities not B cell-associated (*n* = 2). Compared to this study, we found no patients with concordant DLBCL in the BM. However, markedly similar to Margold et al., who reported 7% of discordant findings in PCNSL patients, we found 8.3% of patients with discordant BMB results. The frequency of discordant BM findings in PCNSL is similar to that of systemic DLBCL [2]. Most patients in our study had low-grade lymphoma in the BM, while monoclonal B cells were present in nine of 300 patients, and a lower prevalence than in the study of Brandt et al., who found 8/51 PCNSL patients with monoclonal B cells in BM and 4/51 with low-grade lymphoma [25]. The monoclonal B cells’ prevalence did not differ from the general population, where a variable prevalence was reported (0.12–18.2%) [26]. Furthermore, several studies identified the presence of a concomitant monoclonal B cell small-size population in the BM and peripheral blood as a sign of subclonal, systemic non-CNS disease in CNS lymphoma patients [25, 27, 28]. Interestingly, most of these studies that reported inconsistent findings in BM and tumor-related B cells outside of CNS did not observe systemic relapse of PCNSL [25, 27, 28]. However, Margold et al. reported one case with low-grade lymphoma and relapse of DLBCL in cervical lymph nodes [2]. Future studies are needed to determine the actual value of monoclonal B cells in peripheral blood and BMB in patients with presumed PCNSL.

Although there are concerns that corticosteroids might affect and decrease the value of diagnostic PET-CT, extensive prospective studies in lymphoma patients are lacking [29]. One retrospective study on 178 newly diagnosed patients with aggressive B cell lymphoma found no decreased yield of PET-CT results in patients receiving corticosteroids [29]. When excluding patients with missing information on steroid usage, 87.9% of our population received corticosteroids at some point during the diagnostic workup. In the study of Bertaux et al., corticosteroids were given after brain biopsy but before PET-CT in 105 patients (81%) with a median time of 16.5 days [24]. Only ten patients with concomitant systemic disease among 130 patients with presumed CNS lymphoma were found in this study. However, regarding systemic disease, the authors found no difference in true positivity rates between the few patients not treated and most patients treated with corticosteroids [24]. Contrary, the authors only found that brain PET-CT scans were more likely to be negative in patients receiving corticosteroids for more than one week following a brain biopsy. As an MRI of the brain is recommended before and after brain biopsy and PET-CT is often performed prior to biopsy to differentiate CNS lymphomas from other brain tumors, this seems not to be an issue [30]. Corticosteroids are known to have lymphotoxic effects and should be avoided whenever clinically possible [1]. Due to possible life-threatening mass effects and edema, corticosteroids should be used when necessary to prevent neurologic deficits [1]. The diagnostic results would probably not be impaired following the brief administration of corticosteroid therapy due to the low risk of having BM involvement as the only systemic presentation of CNS lymphoma. Performing diagnostic procedures within a short period, including whole-body PET-CT within a week following a brain biopsy, is highly recommended [6, 7].

The current study’s limitations are primarily due to its retrospective nature, making it impossible to extend the data beyond what is preregistered. Moreover, a significant number of patients were excluded in the early part of the inclusion period screened because PET-CT was not a part of the routine initial staging of patients with CNS lymphoma before the year 2006/2007. Furthermore, BMB was not performed in some patients due to refusal or poor performance status, especially in those patients not fit for intensive therapy. This is the most extensive study to date that analyzed data from patients with CNS lymphoma for whom both FDG-PET scans and BMB results were available. Our findings strongly suggest that BMB can be omitted as part of staging procedures in patients with presumed CNS lymphoma of DLBCL histology and no signs of systemic lymphoma outside of CNS on PET-CT scans, as the risk of overlooking a patient with concordant lymphoma in the BMB is less than 1%.

## Conclusion

This study aims to improve diagnostic workup in patients with CNS lymphoma, spare patients from unnecessary painful BMB, and ensure optimal use of healthcare resources. Based on the analysis of 300 patients with CNS lymphoma and no lymphoma outside of CNS on PET scans, we found 8.3% of discordant findings in the BMB, which did not influence further management of these patients. Contrary, we did not find any patients with DLBCL involvement in the bone marrow. Consequently, we suggest that BM biopsy can be safely omitted from the diagnostic workup in patients with presumed PCNSL and negative PET scans.

## Supplementary information

ESM 1 Suppl. Fig. 1 Kernel density plot of Bayes regression of the risk of bone marrow involvement in patients with central nervous system lymphoma using uninformative and informative priors. Vertical dashed black lines represent the mean of densities, vertical black line marks the risk with 95% cumulative probability, and vertical dashed gray line marks the risk with 99% cumulative probability. (PNG 208 kb) (PNG 208 kb)

High resulotion image (TIF 1663 kb)

## Data Availability

The datasets generated during and/or analyzed during the current study are available from the corresponding author on reasonable request.
