# Supplementary figures and images for: Bone marrow biopsy can be omitted in the diagnostic workup of CNS lymphoma of DLBCL origin: a population-based retrospective study in the PET-CT era
Source: Ann Hematol. 2023 May 29;102(7):1897–905. doi: 10.1007/s00277-023-05282-7 (PMC10261253; doi:10.1007/s00277-023-05282-7)

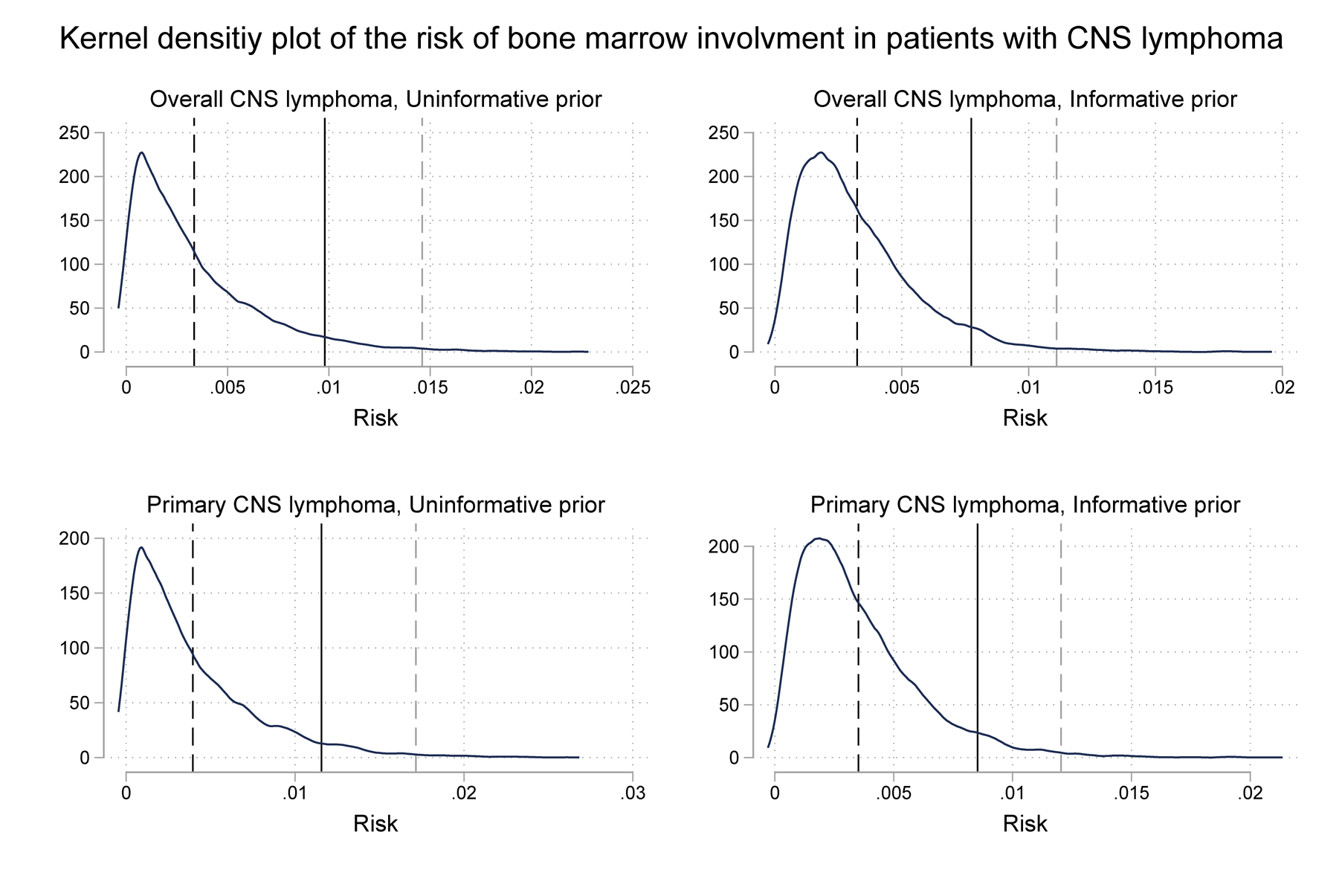

Supplement: Supplementary file 1 — Suppl. Fig. 1 Kernel density plot of Bayes regression of the risk of bone marrow involvement in patients with central nervous system lymphoma using uninformative and informative priors. Vertical dashed black lines represent the mean of densities, vertical black line marks the risk with 95% cumulative probability, and vertical dashed gray line marks the risk with 99% cumulative probability. (PNG 208 kb) (PNG 208 kb) [file 277_2023_5282_Fig2_ESM.png]

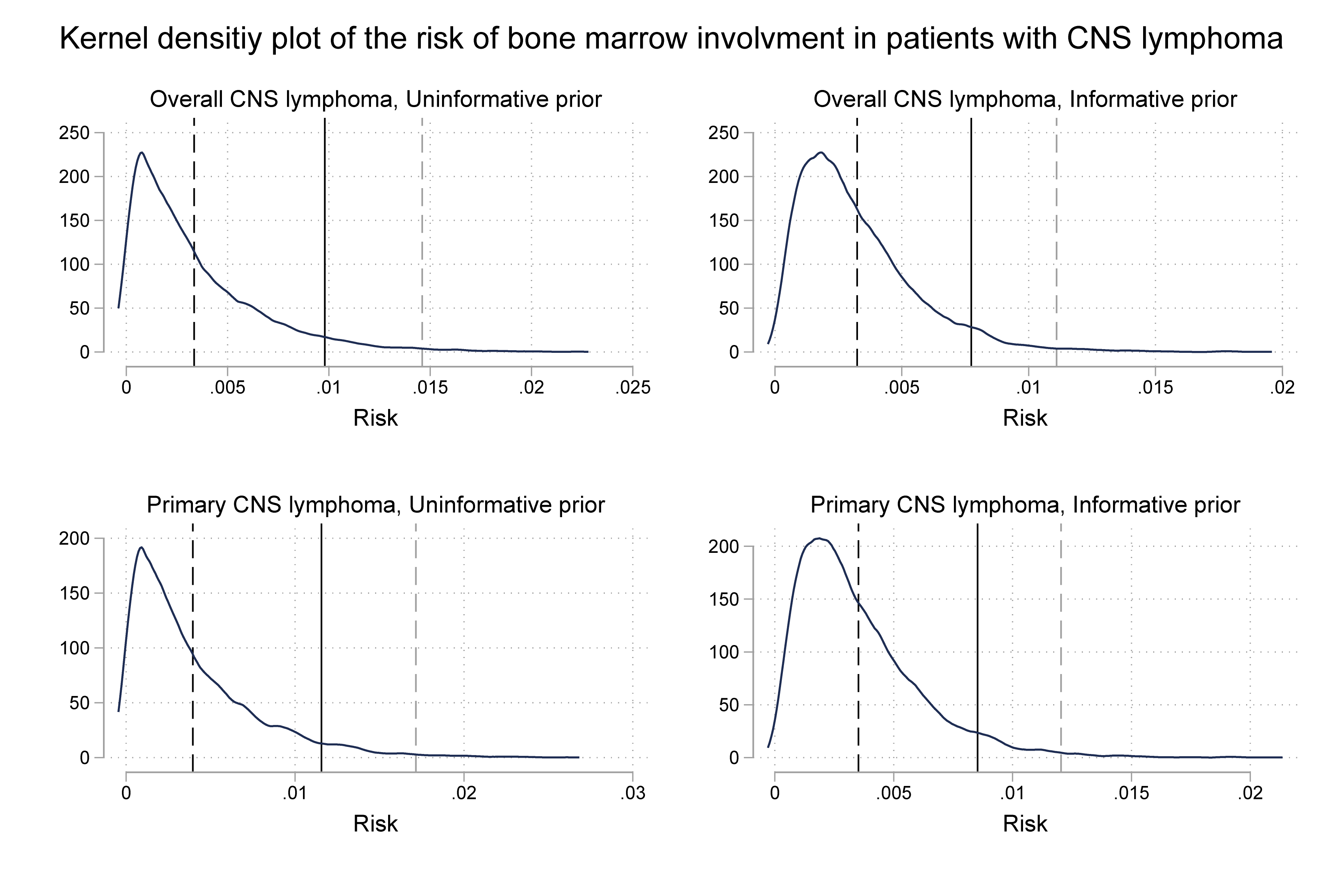

Supplement: Supplementary file 2 — High resulotion image (TIF 1663 kb) [file 277_2023_5282_MOESM1_ESM.tif]
